# Editorial: Alterations of Vestibular Function in Cochlear Implantation

**DOI:** 10.3389/fneur.2021.740690

**Published:** 2021-09-24

**Authors:** Anandhan Dhanasingh, Ingo Todt, Louis Hofmeyr

**Affiliations:** ^1^MED-EL (Austria), Innsbruck, Austria; ^2^Department of Otolaryngology, Head and Neck Suregry, Bielefeld Clinic, Bielefeld, Germany; ^3^Stellenbosch University, Stellenbosch, South Africa

**Keywords:** vestibular function, vestibular testing, cochlear implantation, round window and cochleostomy electrode insertion, vestibular testing in children

Cochlear implantation is a widely accepted treatment option in restoring hearing in subjects of all age groups with profound or partial sensorineural hearing loss. The cochlear implant (CI) electrode array is placed inside the scala-tympani (ST) to electrically stimulate the neural elements, which is linked to the auditory pathway reaching the cortex to complete the hearing restoration process. The ST is filled with a sodium-rich fluid called perilymph, which is highly conductive and facilitates the spread of electrical impulses coming out of the electrode array to inside the ST and possibly other neighboring structures. The scala media (SM) is a small compartment seen right above the ST, separated by the basilar membrane (BM), as seen in the mid-modiolar section, and is filled with potassium rich endolymph. The SM is connected directly to the vestibular portion of the inner ear and any disturbance to the BM and SM during the CI electrode insertion process can disturb the vestibular function. The SM and the BM could be disturbed by the surgical approach of electrode array insertion (round window membrane vs. promontory drilling of cochleostomy) and/or the stiffness along with the length of the CI electrode array that goes inside the ST. Evaluation of the vestibular function involves sensitive testing methods that is often challenging in children. Any disturbance to vestibular function following the CI surgery could negatively affect the patients by causing symptoms of dizziness, feeling off-balance, disorientation, or falling or stumbling and in their overall quality of life (QoL). Therefore, it was the initiative of the editors to set-up this special issue entitled “Alterations of vestibular function in cochlear implantation” inviting researchers to submit their clinical findings along this topic.

This editorial summarizes the key findings of the manuscripts submitted by researchers in response to the invitation, which underwent peer-review process and published successfully. Tsukada and Usami reported a reduced risk of damage to vestibular function using the round window approach in CI surgery with flexible lateral wall electrode arrays in contrast to promontory drilling of a bony cochleostomy and using pre-curved electrode array types. Their findings were supported by Koyama et al. who had similar outcomes in a pediatric population. They went on further to report that even using the extended round window approach of electrode array insertion could affect the vestibular function negatively as evaluated by cVEMP (caloric vestibular evoked myogenic potential). cVEMP is used to assess the patient's balance function by evaluating their saccular function. Wang et al. reported that children with enlarged vestibular aqueduct (EVA) are more likely to have preserved saccular and utricular functions after CI surgery than children with “normal” ear anatomy due to less pressure-related damage to those structures as a result of the EVA. It is generally assumed that the length of the electrode array that is inserted inside the cochlea could also affect the vestibular function. Weinmann et al. reported that the reduced probability of vertigo when using flexible electrodes was not clearly observed nor of negative influence of the electrode insertion length. Rasmussen et al. made an interesting report on the vestibular dysfunction reaching a plateau 4-months post-operatively after which there was no further deterioration or improvement in vestibular function. The long-term effect of the biological stressors on the degradation of vestibular function is still to be understood and therefore long-term follow-ups are necessary to validate the findings of Rasmussen et al. Guan et al. reported no difference in vestibular dysfunction in unilateral and sequentially implanted bilateral subjects following CI. The video head impulse test (v-HIT) is a quick, non-invasive, and relatively cheap test to evaluate vestibular function compared to the caloric test. The other key reason to consider using v-HIT is its ability to test in high-frequency range which is effective enough in testing the body and gaze stabilization. Bassaletti et al. evaluated the effectiveness of using v-HIT to select CI-candidates that require caloric testing before cochlear implantation. Vestibular implants (VI) are currently under research to understand the safety and effectiveness of VI in restoring the vestibular function in patients with conditions like bilateral vestibulopathy and Meniere's disease. Montesdeoca et al. reported that the electrically evoked cVEMPs can be present after cochlear and vestibular stimulation. They claim that by measuring the cVEMP following the direct electrical stimulation of the vestibular portion with VI, the vestibular elements can be stimulated safely. Sonsa-Duranowska et al. showed that hearing preservation techniques in cochlear implantation are connected with vestibular protection, but the risk of vestibular damage is never eliminated completely. QoL in older adults with a history of loss of balance and in patients with a history of Menière's disease after cochlear implantation is an interesting topic. Rogers citing existing literature, reported that the risk of falling in older adults after CI is high and urges proper testing methodology to assess their vestibular function before and after the CI surgery. Lassaletta et al. reported that the hearing results and QoL benefits perceived by CI candidates with Menière's disease is similar to regular CI candidates. They further reported that patients who undergo simultaneous CI and labyrinthectomy may experience worse QoL. Li et al. have nicely photographed the vestibular space and the nerves associated with the vestibular organ using micro-computer tomography (μCT) and high radiation synchrotron phase-contrast images. They report that drilling of a cochleostomy may disturb vestibular organ function by injuring the endolymphatic space and disrupting fluid barriers. The saccule is at particular risk due to its proximity with the surgical area and may explain immediate and long-term post-operative vertigo. Round window insertion may be less traumatic to the inner ear; however, it may affect the vestibular receptors. Their claims are nicely supported by the reports of other researchers mentioned above.

Overall, testing the vestibular function periodically for many years following CI surgeries would help the CI community to learn more about the effect of CI surgery and other factors on the alteration of vestibular function. As editors of all these articles, we would like to encourage the readers to take their time to read these articles and to update their knowledge on these topics.

## Author Contributions

All authors listed have made a substantial, direct and intellectual contribution to the work, and approved it for publication.

## Conflict of Interest

AD is employed by MED-EL as the Head of Translational Science Communication. The remaining authors declare that the research was conducted in the absence of any commercial or financial relationships that could be construed as a potential conflict of interest.

## Publisher's Note

All claims expressed in this article are solely those of the authors and do not necessarily represent those of their affiliated organizations, or those of the publisher, the editors and the reviewers. Any product that may be evaluated in this article, or claim that may be made by its manufacturer, is not guaranteed or endorsed by the publisher.

